# A new perspective on cutaneous leishmaniasis—Implications for global prevalence and burden of disease estimates

**DOI:** 10.1371/journal.pntd.0005739

**Published:** 2017-08-10

**Authors:** Freddie Bailey, Karina Mondragon-Shem, Peter Hotez, José Antonio Ruiz-Postigo, Waleed Al-Salem, Álvaro Acosta-Serrano, David H. Molyneux

**Affiliations:** 1 NTDs, Liverpool School of Tropical Medicine, Pembroke Place, Liverpool, United Kingdom; 2 University of Edinburgh Medical School, Edinburgh, United Kingdom; 3 Department of Parasitology, Liverpool School of Tropical Medicine, Pembroke Place, Liverpool, United Kingdom; 4 National School of Tropical Medicine, Baylor College of Medicine, Houston, Texas, United States of America; 5 World Health Organization, Geneva, Switzerland; 6 National Centre for Tropical Diseases, Saudi Ministry of Health, Riyadh, Kingdom of Saudi Arabia; 7 Department of Vector Biology, Liverpool School of Tropical Medicine, Pembroke Place, Liverpool, United Kingdom; Hebrew University-Hadassah Medical School, ISRAEL

## Introduction

This article considers the current public health perspective on cutaneous leishmaniasis (CL) and its implications for incidence, prevalence, and global burden of disease calculations. CL is the most common form of leishmaniasis and one of a small number of infectious diseases increasing in incidence worldwide [1] due to conflict and environmental factors in the Middle East (“Old World”) and the Americas (“New World”)—regions where it is most prevalent. Recently, the disease has reached hyperendemic levels in the conflict zones of the Syrian Arab Republic, Iraq, and Afghanistan while simultaneously affecting refugees from those regions [2]. Nevertheless, CL is not seen as a priority for policymakers because it is not life limiting. This is evidenced by a lack of commitment in recent years to preventive campaigns and patient provision (limited diagnostic capacity, knowledge of treatment, drug availability) in a number of endemic countries [3].

## Expanding the spectrum of CL disease

Cutaneous leishmaniasis is characterized by the active infection of *Leishmania* spp. and its accompanying lesions, which classically evolve from papules and nodules to plaques and ulcers; we term this the active phase of CL. These lesions commonly self-heal in the absence of treatment after a variable amount of time (usually months) [4]. Importantly, the residual scarring that follows the resolution of active CL infection in all cases is not currently recognized as part of the spectrum of CL disease. We term this scarring “inactive CL” to convey parasitological inactivity of lesions rather than sterile immunity; indeed, in a small number of cases, lesions may contain a focus of parasites [5], although it is yet to be demonstrated if these are involved in further disease transmission [6].

Notably, while inactive CL is not currently recognized within the spectrum of CL disease, other, less common sequelae of CL are. For example, the mutilating mucocutaneous leishmaniasis (MCL) is widely considered a long-term sequela of CL, despite occurring almost exclusively in the Americas region and in only a small proportion of overall CL cases [7]. We therefore advocate for CL to be viewed as a disease of 2 phases (active and inactive) followed by a variable third phase (MCL) and wish to further discuss why this expanded view of CL disease is important.

In common with other neglected tropical diseases (NTDs) with prominent cutaneous manifestations such as onchocerciasis, leprosy, yaws, scabies, and Buruli ulcer, CL is damaging socially and deeply stigmatizing [8]. However, social stigma in leishmaniasis has also been shown to reinforce poverty in affected individuals and thus is of great concern [9]. Notably, it is the lasting aspect of inactive CL (scarring) that generates this considerable stigma; in this sense, stigma in CL is independent of a patient’s microbiological status in endemic communities. This is evidenced by many local terms that equate CL specifically with its scarring form (e.g., “the scar will remain forever” in the Kingdom of Saudi Arabia, “mountain leprosy” in the Amazon region, “Aleppo evil” in Syria, and “trace” in Yemen) [3,10], underlining the importance placed on inactive CL by those affected by the disease.

Moreover, there is known to be a continuation of psychological morbidity with the scarring that ensues post CL infection (both treated and self-healing). This is unsurprising, as epidemiological studies show that approximately 50% of CL lesions are located on the face [1], and lesion visibility is an important risk factor for depression in dermatological conditions. Indeed, the rates of comorbid depression associated with inactive CL may equal if not exceed those of active disease [11,12]. The quality of life of patients is also significantly impaired relative to control groups, and in some cases, this is equivalent to the impairment found in active disease [11,13]. Overall, inactive CL represents a substantial disease burden extending beyond active CL infection. To recognize the extent of the impact of CL on the lives of patients, it is therefore important to recognize that its burden of disease does not end upon resolution of active infection.

Lastly, while both active and inactive forms of CL can be unsightly, the residual scar of inactive CL is hard to remove cosmetically, and thus in the vast majority of cases, scarring is permanent and lifelong. As a result, there is a much greater number of “inactive” CL patients in the world than “active” cases. How CL is viewed as a disease therefore has important and direct implications for how incidence (number of new cases of CL per year) and prevalence (total number of cases of CL) is both reported and estimated. In turn, this then impacts the overall burden of disease as measured by Disability Adjusted Life Years (DALY) estimates for CL, which are based upon prevalence. These aspects will now be further discussed in turn.

## Discrepancies in incidence and prevalence figures

In Table 1, we display figures from various sources that provide information on the global incidence and prevalence of CL, upon which, policy and burden of disease (DALY) estimates have been based. Notably, some CL figures (including WHO and Global Burden of Disease [GBD]) include MCL cases, although these are likely to only represent a small proportion of the overall CL case load (about 5%) [7].

**Table 1:** Reported and estimated incidence and prevalence of cutaneous leishmaniasis, 2002–2015.

Author	Study year	Reported		Estimated	
		Incidence	Prevalence	Incidence	Prevalence
Mathers et al. [14]	2002	-	-	1,157,000	2,157,000
Alvar et al. [15]	2002–2009	214,036	-	1,213,300	-
WHO WER [16]	2014	154,649*	-	-	-
WHO GHO [17]	2005–2015	187,855* (mean)	2,066,410* (11 years)	-	-
GBD 2010 [18]	2010	-	-	-	10,000,000
GBD 2013 [19]	2013	-	-	-	3,914,800*
GBD 2015 [20]	2015	-	-	-	3,895,900*

N.B. The studies below the dotted line (**...**) refer to Global Burden of Disease (GBD) studies conducted by the Institute of Health Metrics and Evaluation (IHME)

*MCL included

**Abbreviations:** GBD, Global Burden of Disease; GHO, Global Health Observatory; WER, Weekly Epidemiological Record

Overall, estimates of CL incidence have increased from 1.1 to 1.2 million cases per year from 2002–2009 [14,15], which is 6- to 10-fold higher than reported incidence data. Larger increases in incidence have, however, been recently noted at regional levels [2]. On the other hand, estimates of CL prevalence have almost doubled from 2.1 million cases in 2002 [14] to nearly 4 million cases in the 2015 GBD study [20]. Whilst increases in CL prevalence are to be expected in light of the lasting nature of CL sequelae and its increasing incidence, such estimates seem unrealistically small. For example, this latter figure of 3.9 million prevalent cases from the 2015 GBD study represents only twice the sum of 11 years of reported incidence from WHO (2005–2015) [17] and only 3 times the previously estimated annual global incidence [15].

From these figures, it is clear that (1) only active disease has been included in incidence calculations, and (2) scarring (inactive CL) is not factored into prevalence estimates. This is apparent because, otherwise, CL prevalence estimates would be significantly higher. As CL is not a life-limiting condition, we would also expect prevalence estimates to have increased consistently throughout the study period. In the past 11 years (2005–2015) alone, 2 million new cases have been reported by WHO [17]. Given that the number of estimated cases varies from 6- to 10-fold higher than reported cases [7,15,16], the actual numbers of inactive CL patients could be between 12–20 million (assuming no deaths). However, the life expectancy of patients with scarring CL is likely to exceed the 11 years represented by WHO’s figures. Applying a highly conservative life expectancy of 20 years for affected individuals with inactive CL, it is possible that upwards of 40 million inactive CL patients are currently living with the aforementioned psychosocial consequences of past infection.

## Implications for global burden of disease (DALY) calculations

It is evident that CL is being viewed from a purely parasitological perspective in current prevalence estimates. However, as mentioned, prevalence is the major determinant of the Years of Life with Disability (YLD) component of DALY calculations. This implies that when patients are no longer positive for *Leishmania* spp., they are considered to not be affected by the disease in such calculations. If the scarred CL patients were included in the prevalence estimates of GBD studies (approximately 40 million cases) instead of simply those with active infection (approximately 4 million cases) [20], then the estimated burden of CL disease would be increased by a factor of 10. Such findings have important implications with respect to prioritizing CL for global disease control and research and development (R&D) needs, including CL drugs, diagnostics, and vaccines [21].

In the 2010 GBD study, leishmaniasis (CL, MCL, and visceral leishmaniasis [VL]) had the largest single-cause disease burden of any NTD [18], yet less than 10% of the overall disease burden was accounted for by CL, because inactive CL was not included in the study. This demonstrates that it is not possible to understand the true burden of CL disease without first recognizing its inactive scarring component. From this, we can extrapolate that the psychological impact associated with other, less prevalent leishmaniases such as post-kala-azar dermal leishmaniasis (PKDL) is equally unrecognized; for example, PKDL is by definition a sequela of VL but has not been included in any estimate of the burden of VL disease to date.

Furthermore, the unlikely finding that leprosy and CL both had among the lowest YLDs of any NTD in the 2015 GBD Study [20] raises questions about the ability of such estimates to capture the important stigmatizing nature of such diseases. Overall, the purpose of the DALY is to recognize and compare the morbidity and the mortality of patients with a range of conditions. However, for a lifelong stigmatizing disease such as CL (as well as other chronic NTD skin conditions), the current microbiological perspective to disease monitoring leads to a massive underrecognition of the true disease burden of affected individuals in GBD studies and therefore calls into question the validity of such comparisons.

## Conclusion

We conclude that the current view of CL neglects a large majority of patients living with continued stigma and psychological burden postinfection and show that this has major implications for the way global prevalence estimates are generated, which in turn directly impacts burden of disease calculations. Of particular concern is the long-term impact of CL on endemic and conflict-affected countries and especially the role of CL in promoting poverty in such settings, which has not been addressed. The possibility that up to 40 million people suffer from the long-term stigmatizing effects of inactive CL scarring suggests that the disease is a large-scale global health problem. Appropriate revision of current CL estimates is therefore critical in order to better prioritize this neglected skin disease for global efforts and R&D needs.
